# Shrubby cinquefoil (*Dasiphora fruticosa* (L.) Rydb.) mapping in Northwestern Estonia based upon site similarities

**DOI:** 10.1186/s12898-017-0117-0

**Published:** 2017-02-21

**Authors:** Kalle Remm, Liina Remm

**Affiliations:** Institute of Ecology and Earth Sciences, University of Tartu, Vanemuise 46, 51014 Tartu, Estonia

**Keywords:** *Dasiphora fruticosa*, Similarity-based reasoning, Species distribution mapping, Gower’s distance metric

## Abstract

**Background:**

Different methods have been used to map species and habitat distributions. In this paper, similarity-based reasoning—a methodological approach that has received less attention—was applied to estimate the distribution and coverage of *Dasiphora fruticosa* for the region in the Baltic states where grows the most abundant population of this species.

**Methods:**

Field observations, after thinning to at least 50 m interval, included 1480 coverage estimations in the species presence locations and 8317 absence locations. Species coverage for the 750 km^2^ of directly unobserved area was calculated using machine learning in the similarity-based prediction system Constud. Separate predictive sets of site features (e.g. land cover, soil type) and exemplar weights were calibrated for spatial partitions of the study area (probable presence region, unclear region, proved absence region). A modified version of the Gower’s distance metric, as used in Constud, is described.

**Results:**

The resulting maps depicted the predicted coverage, the certainty of decision when predicting presence or absence, and the mean similarity to the exemplar locations used while predicting. Coverage prediction errors were smaller in the unclear partition—where the species was mostly absent—than in the probable presence partition, where coverage ranged from 0 to 90%.

**Conclusions:**

We call for methodological comparisons using the same data set.

## Background

Detailed distribution data are needed in order to monitor changes in species’ distributions, for conservation, territorial planning, and species and habitat management, but it is impractical and expensive to conduct detailed field observations over large areas. For a detailed distribution map that covers hundreds of square kilometres, knowledge regarding the limiting and the favourable factors that affect the target species is required. The likelihood of a species being present or absent at unobserved locations can then be predicted using a statistical model [1–4], or alternatively, according to similarity of exemplar sites [5]. Exemplars are the cases selected out from a training data set by machine learning or by expert decision. Maps of a species estimated distribution are also important for further monitoring efforts, since predictions help to identify areas in need of urgent future sampling [6, 7].

Similarity-based—also known as case-based—reasoning is an alternative to statistical regression models and classification methods [8]. The use of similarity-based reasoning is widespread in the fields of image recognition [9], medicine [10], web and text mining [11], engineering [12], meteorology [13], site classification [14], and other subjects where large databases of previous cases exist and case studies dominate over highly formalized rules. Case-based systems reuse previous experiences at a low level of generalisation, do not produce models based on generalized statistical relationships and can be continuously updated with new knowledge, as new cases may be added to the case-base. There is no need to change a model if additional training data becomes available. Similarity-based reasoning is classified as machine learning if an iterative fitting of exemplars, feature weights, and other parameters, precedes the inference.

Similarity-based distribution mapping assumes a species (or other phenomenon) to occur in locations similar to those where the species has already been recorded. The principal difference of case-based reasoning from niche-based distribution models lies in not assuming and applying a niche as a theoretical abstraction. Case-based systems infer directly from the most similar exemplars, not using any theoretical model, except rules how to calculate similarity. Similarity-based methods are rarely used for species distribution and habitat suitability mapping, though some examples exist. T. H. Booth compared the climatic similarity of locations to identify sites suitable for introduction a tree species outside its natural range [15]. Carpenter et al. introduced DOMAIN—a similarity-based algorithm for modelling potential distributions of plant and animal species [16]. Clark compared characteristics of black bear sites with the variate mean values of all visited sites using the Mahalanobis distance statistic to map habitat suitability for the bear [17]. Skov introduced a software application for creating site similarity-based plant species distribution maps [18]. De Siqueira et al. mapped summary of environmental distances as similarity measures in a 16-dimensional environmental space to the known occurrence point of a rare plant species [19]. Remm and Remm created maps depicting the similarity of each location to the observed presence and absence sites of ten orchid species using software system Constud [20]. The methodological advancement compared to the previous similarity-based habitat mappings was machine learning of optimal weights for site features and for observed locations.

Shrubby cinquefoil (*Dasiphora fruticosa* (L). syn. *Potentilla fruticosa*, Rosaceae) is a perennial flowering shrub, mainly known for being decorative cultivar. This species has widespread natural populations in mountainous regions of Asia and across North America (except in the south); its distribution in Europe is more fragmentary, being found only in Pyrénées, Maritime Alps, Rhodope Mountains, Crimea, Ireland, Great Britain, Öland and Gotland (Sweden), north-western Estonia, and one location in Latvia [21, 22]. Shrubby cinquefoil cultivars differ genetically from natural Northern European populations [21]. Shrubby cinquefoil probably had a continuous distribution over the present boreal and nemoral zones after the Late Glacial Maximum (c. 13,000–10,000 years ago), but was eliminated from large areas owing to soil leaching, peat accumulation, and forestation [23, 24]. More recently, ploughing probably destroyed many populations.

The populations of *D. fruticosa* have mostly patchy spatial pattern owing to vegetative spread by sprawling stems and rare establishment of seedlings under natural conditions [21, 25]. As a field mapping object, shrubby cinquefoil is easy to detect visually, especially from the end of June to September when it is in flower. In Estonia, the bushes are usually about half a metre high, although on rare occasions it can grow to more than a metre in open places, and can even reach more than 1.5 m when leaning on juniper (*Juniperus communis*) bushes.

Shrubby cinquefoil is a protected plant in Estonia. The only sustainable population is between Tallinn, Keila, and Paldiski [21], where it is mainly found on alvar grasslands spread over Middle and Upper Ordovician limestone, and forms the largest natural population in the Baltic states. During the previous decades, urban sprawl around Estonia’s capital Tallinn has encroached upon unique *D. fruticosa* alvars. On residential or industrial building plots, only a few natural *D. fruticosa* bushes have been retained according to our field experience. Although the species population within this area is currently viable, its health and continued existence needs attention and monitoring.

The Estonian Nature Information System, held at the Ministry of the Environment, contained 18 sites where the species was known to be present before this project was started, 15 of which fall within the region covered by this study. The species presence sites registered by the Ministry are publicly available from http://xgis.maaamet.ee/xGIS/XGis?app_id=UU62A. However, it is unclear to what extent these records represent the current distribution in spatial detail, since: (1) absence sites are not recorded; and (2) the spatial intensity of observations used to delineate species occurrence polygons in the national database is unknown.

The aim of this study was to create a detailed similarity-based map of the distribution of *D. fruticosa* in the study area, by combining the site features selected during a previous study [26]. In addition to the map, a novel technological approach, which included study area partitioning, will be proposed for spatially detailed distribution mapping.

## Data

### Study area

**Fig. 1:**
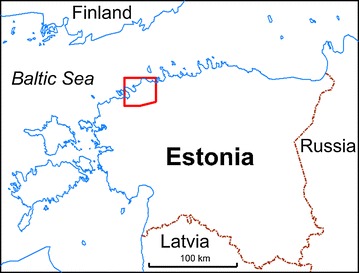
Location of the study area (*red rectangle*). Redrawn from the public wms service http://kaart.maaamet.ee/wms/alus?

The study area covering 819 km^2^ was located at the known *D. fruticosa* natural occurrence area in North-western Estonia (Fig. 1). According to a database of landscape categories, limestone, lacustrine and marine plains, and mires, dominate the study area [27]. The ground elevation above sea level of the study area was up to 58 m, with most (64%) lying between 20 and 40 m. According to the Estonian National Topographic Database held by the Estonian Land Board, forest covers 45%, cultivated land 22%, natural grassland 13%, private yards 5%, unmanaged open land (in this region mainly alvar grassland) 4%, scrubland 2%, and inland waters 1% of the study area. Most residents within the study area live in towns. Approximately 28% (242 km^2^) of the area has no permanent residents; more than half of the territory is sparsely populated with 1 to 100 inhabitants in 497 one square kilometre grid cells.

**Fig. 2:**
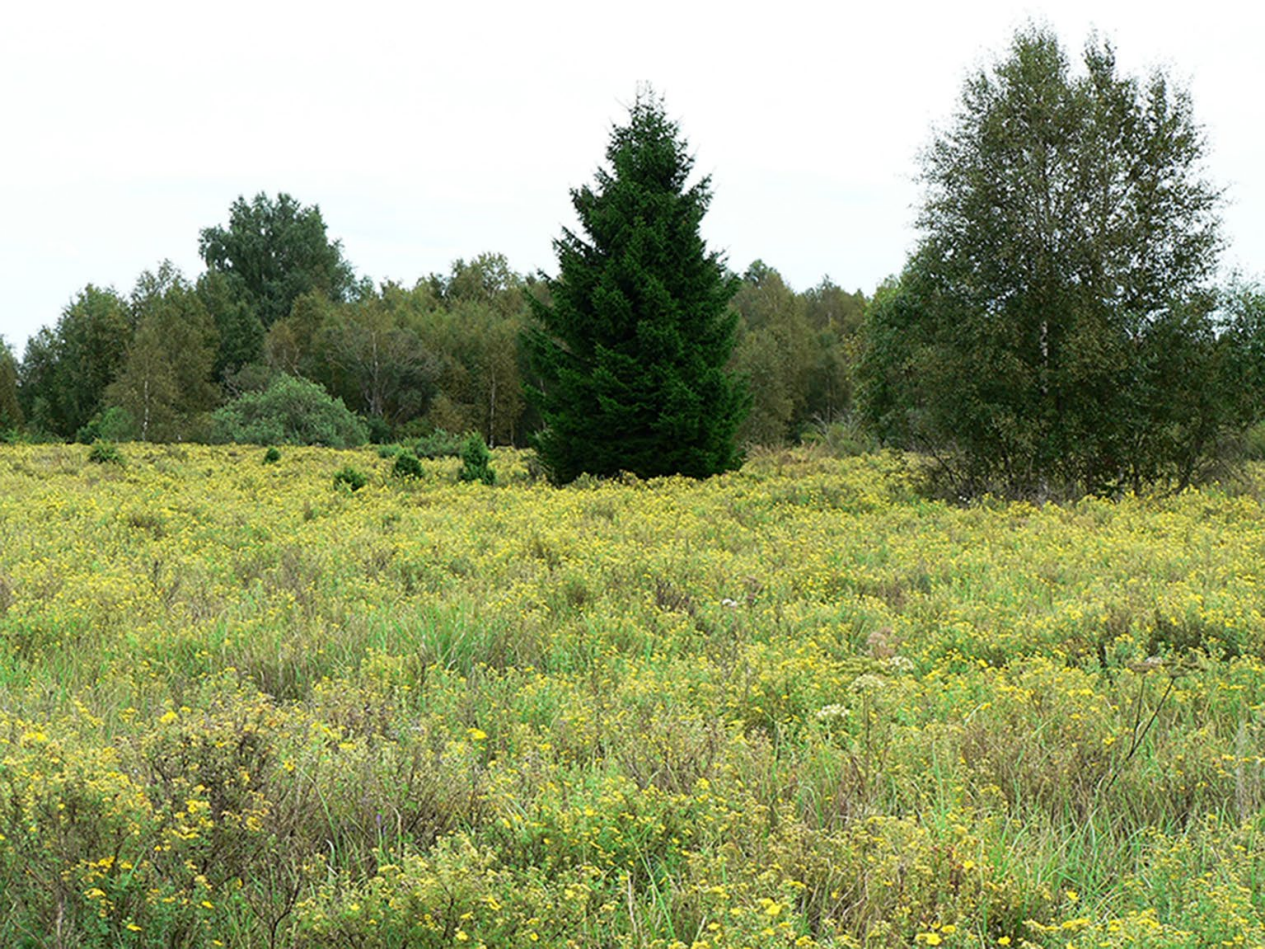
Field of flowering *D. fruticosa* at Vääna Landscape Reserve

**Fig. 3:**
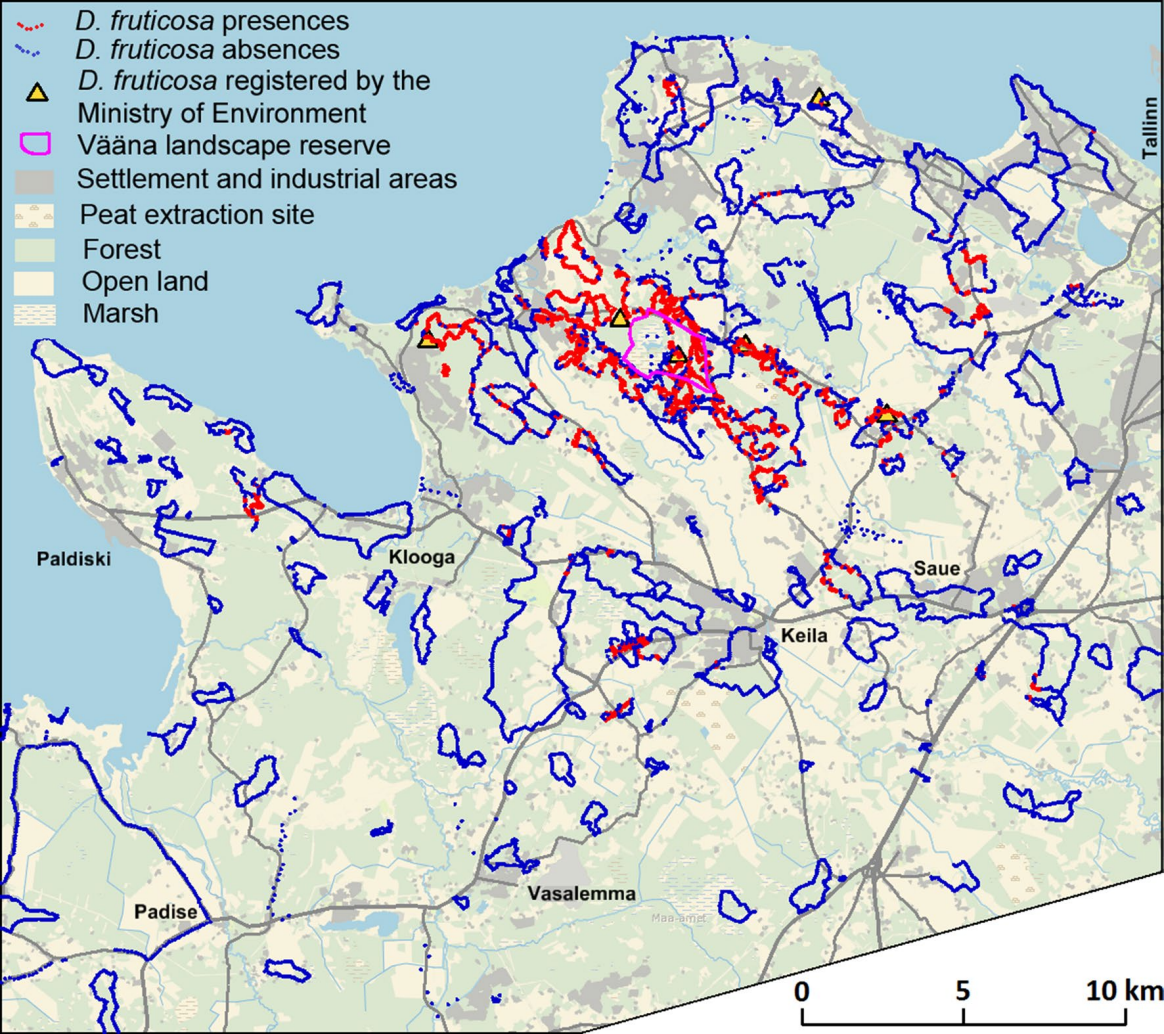
*D. fruticosa* records from this study (presence = *red*, absence = *blue*), presence sites registered by the Ministry of the Environment in the beginning of this project (close locations are depicted by one triangle; the distribution data are public), and the Vääna Landscape Reserve (*magenta*), on a background map from the Estonian Land Board

The study area contains the Vääna Landscape Reserve (4.1 km^2^), created to protect inter alia the alvars where grows the largest natural population of *D. fruticosa* in the Baltic states (Figs. 2, 3). Similar alvar sites can be found elsewhere in western and northern Estonia, but *D. fruticosa* does not grow there.

### Fieldwork

Field observations were made during the summers of 2008–2014 conducting walking tours across the terrain, preferably in regions representing land cover and soil categories, which, according to so far collected field data, could be suitable for the species, but where the density of our observations was lower and the presence or absence of *D. fruticosa* unclear.

The coordinates of the movement tracks, *D. fruticosa* occurrence locations, and deliberately selected typical absent locations were recorded using a Garmin Vista HC+ GPS recorder. *D. fruticosa* coverage percentage within 10 m was visually estimated at each occurrence location. The total length of the observation trails was at least 700 km (Fig. 3). Most (78%) of the study area contains observed sites within a radius of 1 km and 96.4% within a radius of 2 km; although, at the more detailed 100 × 100 m output grid level, only 8.3% (68.3 km^2^) is covered by the direct observations. For the rest of the study area (750.7 km^2^), the predicted occurrence and coverage of the species were calculated according to site similarity (see “Methods” section).

The GPS track recorder automatically stores coordinates along the route at variable interval according to the change in movement direction. The raw data contained 58,842 automatically recorded track points and 5687 actively recorded observation locations (2854 presence sites and 2833 absence sites). The raw records were thinned to ensure a distance of at least 50 m between each accepted site, in order to level out the density of the observations and avoid spatially close records. The track points at more than 50 m from the closest recorded presence location were considered, in addition to the deliberately selected absence exemplar sites, as absence records. Thinning resulted in 8317 absence and 1480 presence locations at a spatial interval of at least 50 m. The species cover estimations in the thinned locations are freely available as an archived dataset [28]. More details regarding the field observations and data thinning are given in [26], which is based on the same data set.

### Data layers and site features

The possible number of numerical features for any geographical location approaches infinity, if to consider different reference time, spatial and thematic generalization levels and neighbourhood extent options for deriving numerical features from data sources. The more indicative site features for mapping the species’ distribution were selected in a previous study based on the same data set [26]. These were: present land cover type (15 categories); historical land cover (6 categories); the most frequent (modal) historical land cover at a radius of 200 m; tussocks dominating or not within a 1 km radius; local soil type (25 categories); the modal soil type within a radius of 200 m; and features describing the spatial autocovariation of the species’ occurrence (the proportion of finds within radii of 100 and 1000 m, the mean coverage within 100 and 1000 m, and the reverse distance-weighted mean coverage).

The land cover and soil data for calculating these features were obtained from Land Board Estonia: the land cover data from the Estonian National Topographic Database, soil data from the Estonian 1: 10,000 soil map. Historical land cover types were digitized from a topographical map surveyed in the second half of the 1930s, the mean coverage and the proportion of presences in neighbourhood are calculated from observed coverages in locations retained after thinning. Site features used for machine learning were read from 10 × 10 m grid layers rasterized from vector format source data. Prediction maps were calculated as 100 × 100 m Idrisi (Clark Labs) raster format grids.

## Methods

**Fig. 4:**
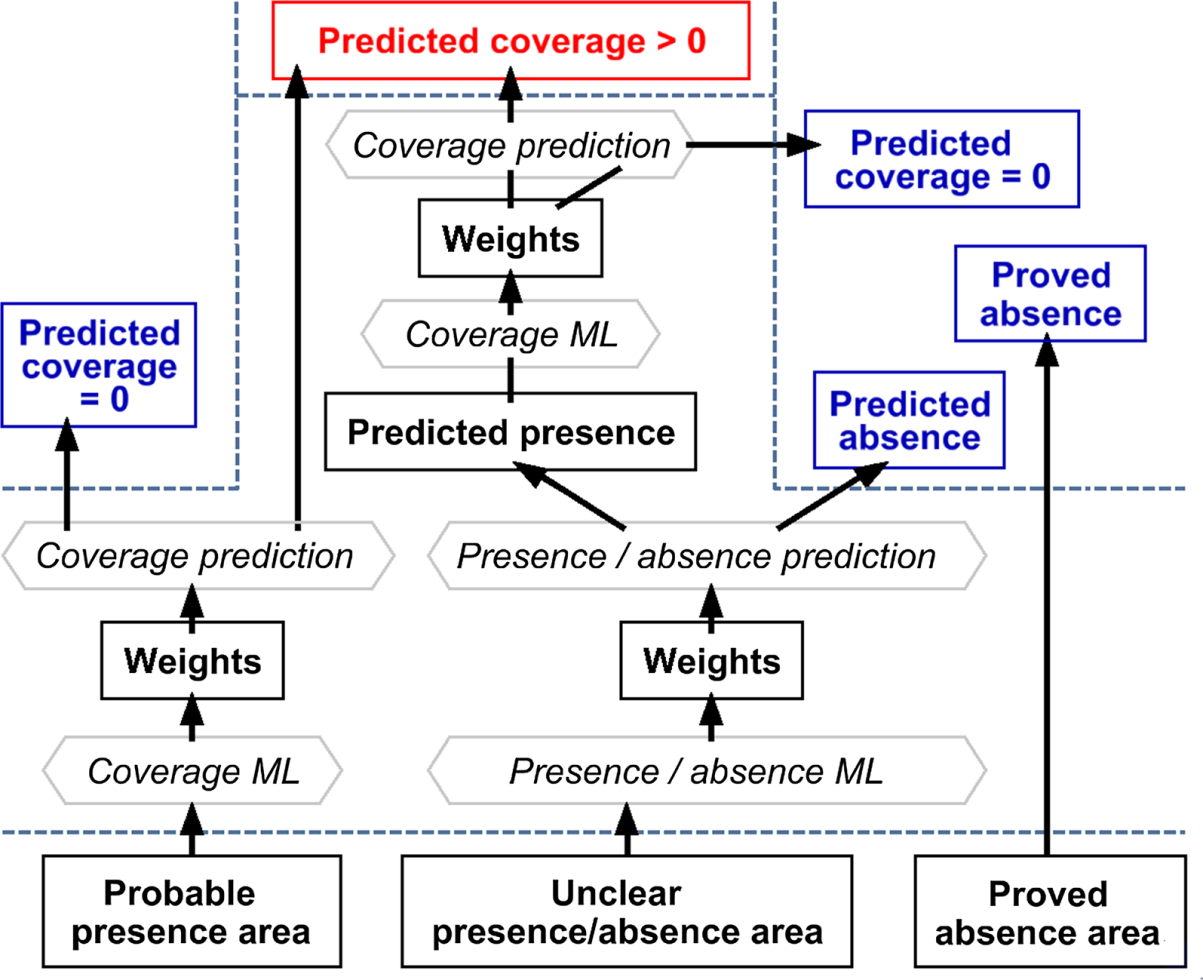
A flow diagram of the similarity-based distribution mapping of *D. fruticosa*. *ML* machine learning in Constud (iterative optimization of weights for features and exemplars), *weights* optimized weights for features and exemplars

*Dasiphora fruticosa* distribution mapping consisted of six stages (Fig. 4). The weights for features and exemplars were separately calibrated using software Constud (see the next paragraph) for stages 3–5.

1. Selecting site features indicative to the presence/absence of the species.
2. Partitioning the study area to the species’ probable presence region, proved absence region and unclear region (Fig. 5).

**Fig. 5:**
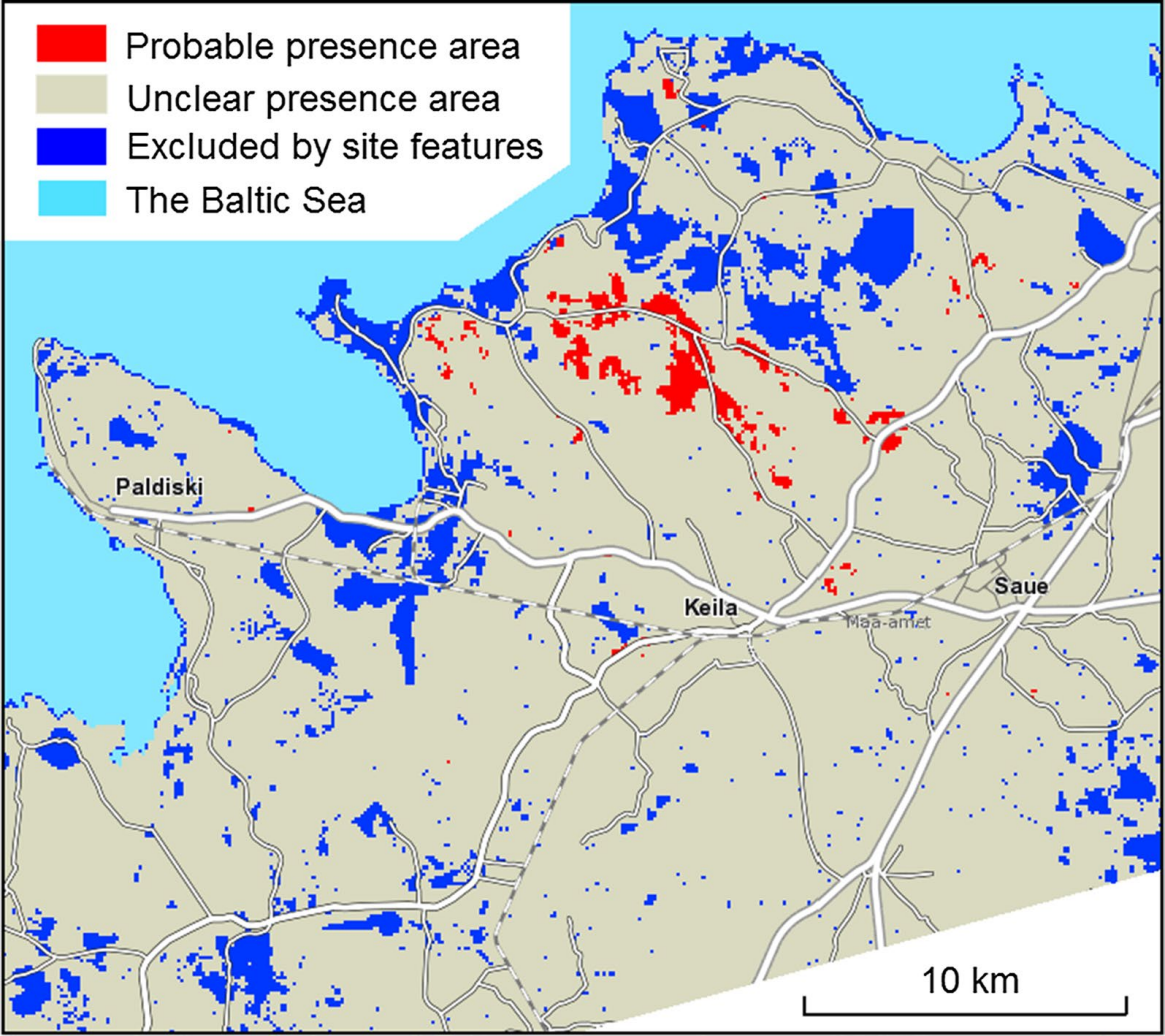
Spatial partition of the study area on a background map from the Estonian Land Board
3. Calculating the expected coverage in the probable presence partition.
4. Calculating the expected presence/absence in the unclear partition.
5. Calculating the expected coverage at each predicted presence site in the initially unclear partition.
6. Overlaying the observed data to the map of predicted values.

The first and second points in this list refer to the stages of the wider research project and are reported in a separate publication [26] where boosted classification tree models were used to compare the value of 60 individual site features at thematically and spatially different levels of generalization as indicators of the species’ presence or absence. The 60 site features were calculated from Estonian land cover database, soil map, a historical map, elevation data, from human population density, and from the species data in vicinity of every location.

The criteria for pre-classifying the study area were as follows. The proved absence partition (41.3 km^2^): more than 1% of observations and no presences; the probable presence partition (11.4 km^2^): values of at least two site features among the four most firm presence predictors indicate the species presence; unclear partition (766.3 km^2^): most of the study area, which is meeting neither of these two criteria. The proved absence partition does not contain any currently known *D. fruticosa* find sites; the probable presence partition contains both presence and absence sites, but presence sites are more frequent. The unclear partition contains predominantly absence records but includes sites potentially suitable for the species.

Calculated values (stages 3, 4 and 5 in the list above) were similarity based estimations. If the site features in a currently predicted site are similar to an exemplar site, then the species coverage in the predicted site is expected to be similar to the species coverage in the exemplar site. Details of the algorithm are given below. Finally the observed coverage records were overlaid to the layer of predicted coverage values assuming the field records to be more reliable than the calculated values.

**Fig. 6:**
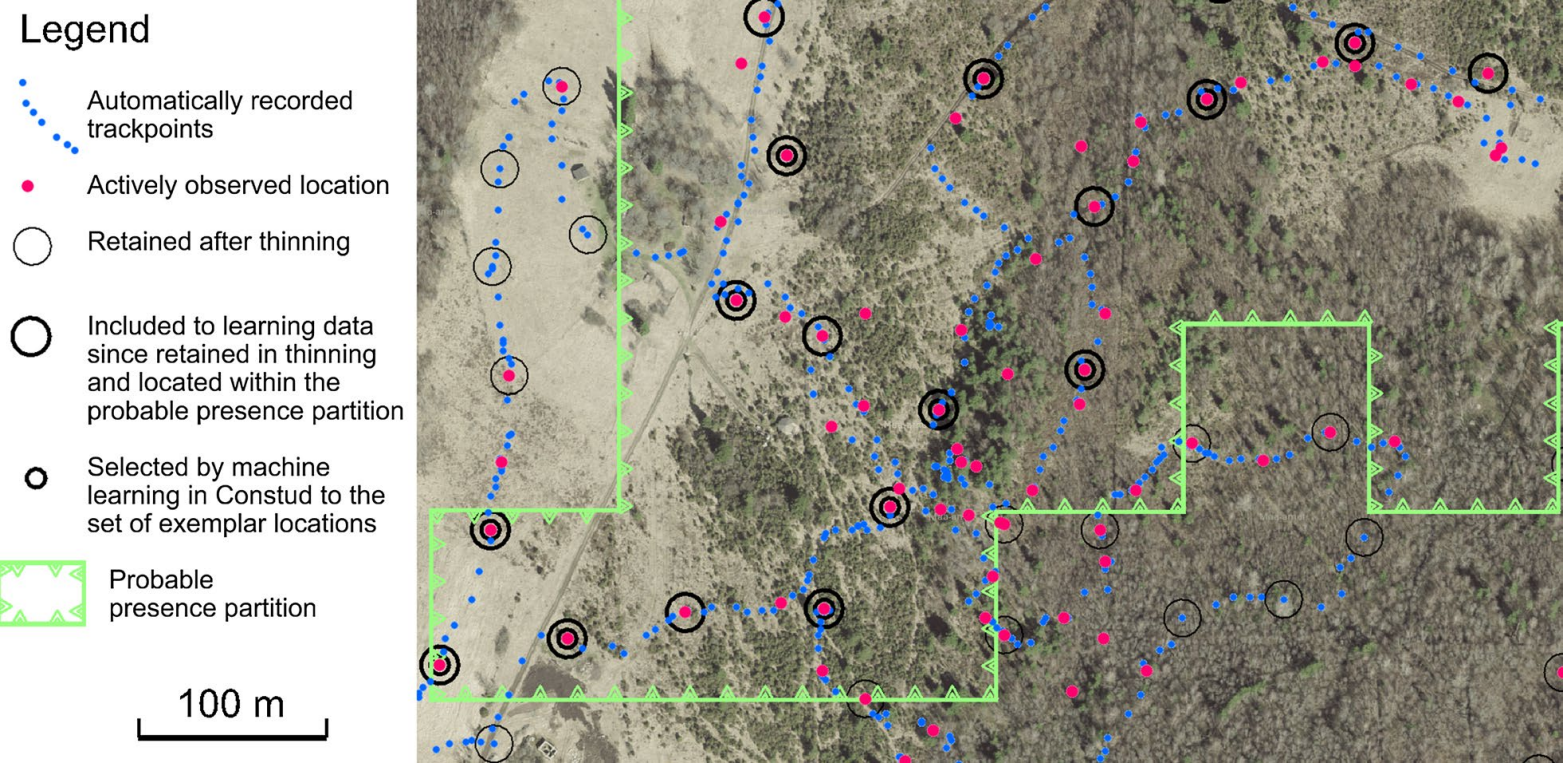
An excerpt from the data as used for predicting *D. fruticosa* coverage in the species probable occurrence partition of the study area. Background—ortophoto from the Estonian Land Board. Only data from locations retained in thinning and located within the probable occurrence partition (a separate set of learning data was used in each partition) were used in machine learning of feature and exemplar weights

Both spatial thinning and machine learning contributed to data reduction. Spatial thinning removes spatially redundant records and machine learning of weights removes redundant learning cases including of which does not reduce prediction error. In addition, only data from the same partition are included since a separate set of learning data was used in each partition (Fig. 6).

We used the software system Constud 3 [29] for machine learning of the optimal weights for features and exemplars, and for the calculation of similarity-based maps for *D. fruticosa*’s occurrence and coverage. Constud as a software system for similarity-based predictions is described in [30]; recent changes compared to the previous version can be found at [31].

**Fig. 7:**
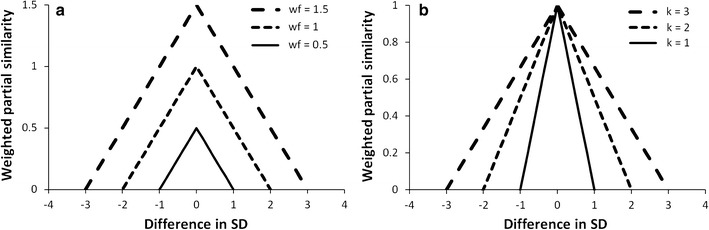
Weighted partial similarity according to the difference in feature values, measured in standard deviations and according to: **a** the feature weight (*k* = 2; *we* = 1), **b** the parameter *k* (*wf* = 1; *we* = 1)

The central operation of the similarity-based reasoning is similarity metering. The Gower’s [32] metric is commonly used for quantifying the distance between two objects. The Gower’s metric uses range standardization to equalize the contribution from each numerical feature. The distance metric in Constud (1) differs from the Gower’s metric, by using the sum of partial similarities *PS* (*Of, Ef*) as weighted by feature weights (*wf*) and exemplar weights (*we*), and by replacing the range standardisation with *k* × *SD* for numerical features (where *k* is the sum of similarity searched for decision—the default value in Constud is 2—SD the standard deviation of the feature, and |*Fe* − *Fo*| the difference in feature values) (1). The partial similarity is calculated between an observation *O* and exemplar *E* regarding only a single feature *f.* Negative partial similarity values are assigned a value of zero. Features with a higher weight have a wider accepted difference in values and have a larger share of the total similarity (Fig. 7).

1 $$PS\left( {Of,Ef} \right) = {\text{wf }} \times {\text{wo }} \times \left( {1 - \frac{{\left| {Fe - Fo} \right|}}{{ k \times {\text{SD}} \times {\text{wf }} \times {\text{we}}}}} \right)$$

This formula (1) is applied for numerical features. When similarity between categories (*Sc*) is considered, the similarity of matching categories is equal to one and of different categories equal to zero (2); otherwise, category-specific similarity values can be assigned by the Constud user and stored in a special database table. We applied the first option in this study.

2 $$PS\left( {Of,Ef} \right) = Sc \times {\text{wf }} \times {\text{we}}$$

Total similarity (TS) between feature vectors is measured as the mean of partial similarities, weighted by the feature and exemplar weights (3). Zero-weight features, i.e. those unsuitable due to temporal limits or missing data, are skipped, as are zero weight exemplars.

3 $$TS\left( {O,E} \right) = \frac{{\mathop \sum \nolimits_{f} \,PS\left( {Of,Ef} \right)}}{{\mathop \sum \nolimits_{f} \,({\text{wf }} \times {\text{we}})}}$$

The prediction fit for a binomial variable (presence/absence) is calculated in Constud as the True Skill Statistic ranging between −1 and +1, zero is the expected value in case of random decisions [33]; for a numerical variable, the objective function is the root mean squared error (RMSE) ranging from 0 to ∞.

Machine learning in Constud involves an iterative search for the best predictive weight sets for the exemplars records and the features to use in similarity-based recognition of the predictable variable. The weights are ready for calculating predictions, either as raster map or as a database object. In addition to the predicted values, Constud enables the user to calculate the mean similarity to the exemplars used for each decision; for nominal dependent variables, the similarity to a given category and the certainty of the decision can also be computed. A low similarity at any location indicates a lack of similar exemplars, and the probable need for additional data collection. A low certainty value means nearly equal similarity of a case to the exemplars of alternative categories; a certainty equal to one indicates that the exemplars used for predicting the dependent variable in this site represent only the predicted category.

Constud settings and results are stored in a SQL Server database, and in principal, can be modified by the user. The main initial parameters for Constud’s learning of the *D. fruticosa* data were: a training sample size of 500; a validation sample size of 1000; the initial amount of similarity used to search for a decision = 5; the number of learning iterations was 200. The proportion of presences and absences was not equalized in the training samples. The grid interval for the output maps was 100 m.

## Results

**Fig. 8:**
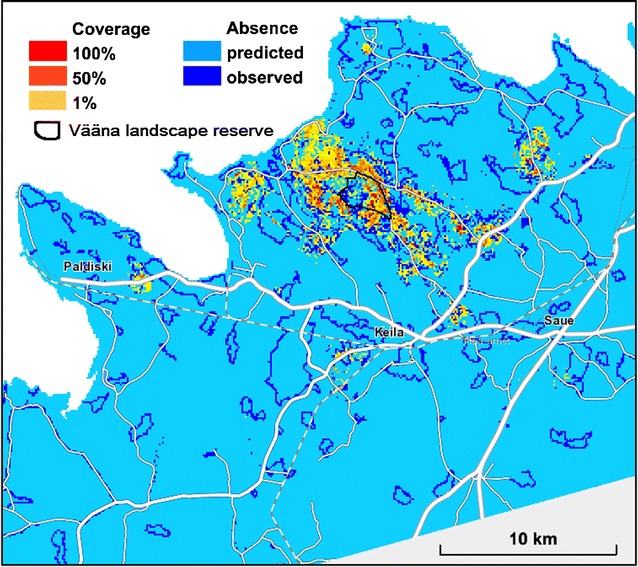
Predicted *D. fruticosa* coverage in the study area on a background map from the Estonian Land Board

**Fig. 9:**
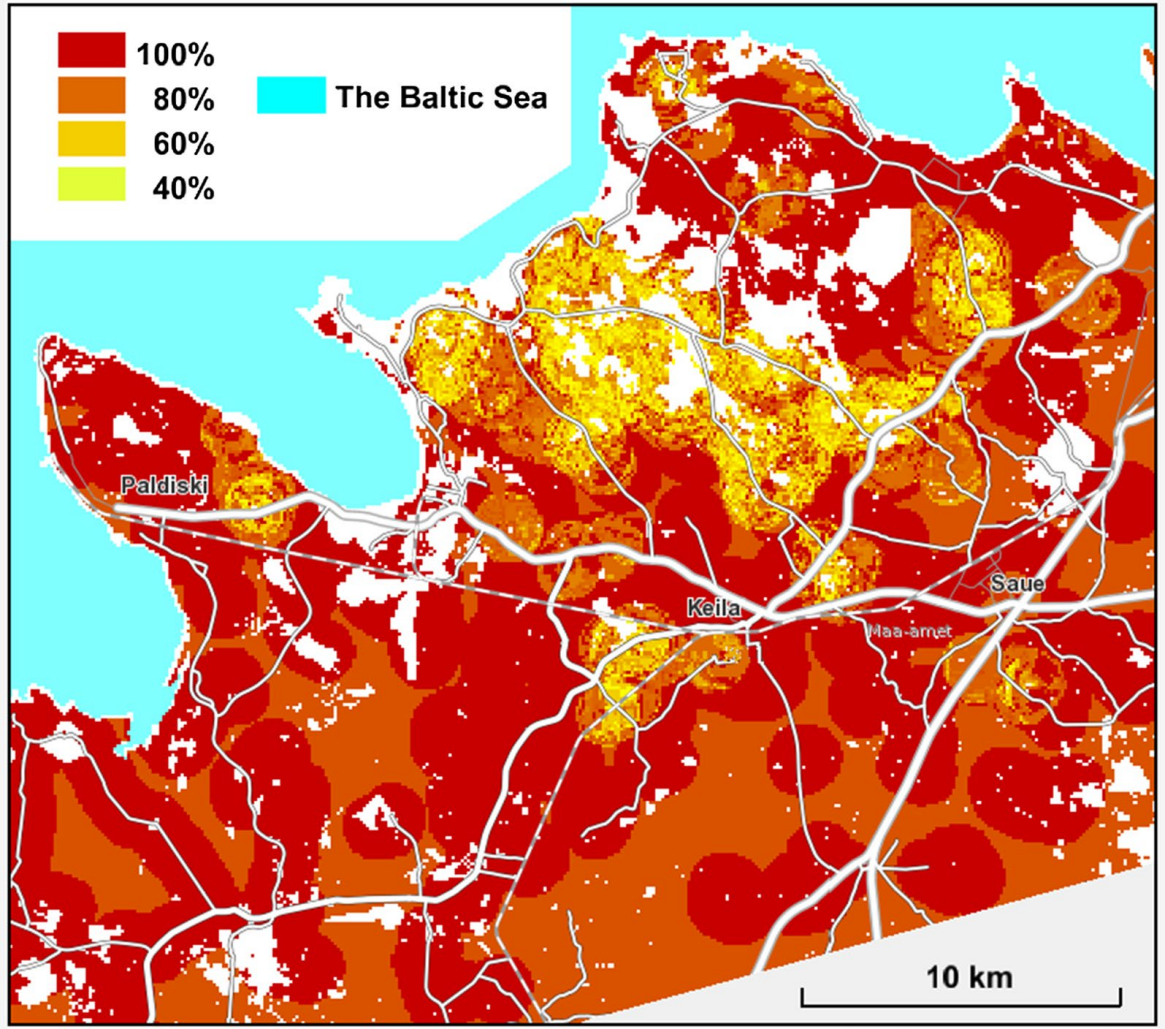
The certainty of decision making in predicting *D. fruticosa* presence/absence in the unclear area on a background map from the Estonian Land Board

The main result was the mapping of estimated *D. fruticosa* coverage in the study area (Fig. 8). The recognition of *D. fruticosa* presence and absence sites within the hitherto unclear partition was not firm: the True Skill Statistic in the validation sample = 0.64. The certainty of decisions was low mainly around registered occurrence sites (Fig. 9); elsewhere the prediction was firmly the species absence.

The predictive set selected by the machine learning included only features that described autocovariation regarding the distribution of *D. fruticosa* (Table 1); i.e. soil and land cover did not support considerably the recognition of species presence sites, when the features describing autocovariation had values (the occurrence and coverage of the species in the neighbourhood was known). A high proportion of presences in the 100 m vicinity was the most reliable occurrence predictor, but was not a firm predictor, as there were always at least some absence records near observed presence sites.

**Table 1:** Machine learned (ML) feature weights of the predictive sets selected in Constud

Feature	Num. of classes	Radius (m)	ML feature weights in predicting		
			Presence/absence in unclear partition	Coverage in	
				Predicted presence sites	Probable presence partition
Present land cover	15	0		0.8	1
Historical land cover	6	0		0.3	0.1
Historical land cover	6	200			0.4
Historical land cover	2	1000			0.1
Soil	25	0			1
Soil	25	200			0.3
Proportion of presences		100	2.9		0.1
Proportion of presences		1000	0.5	0.6	5.5
Mean coverage		100	0.2	2.0	0.2
Mean coverage		1000	0.3		0.6
Distance weighted mean coverage		10,000	1.1		1.8

The three ML sets of feature weights match the machine learning operations in Fig. 4

**Fig. 10:**
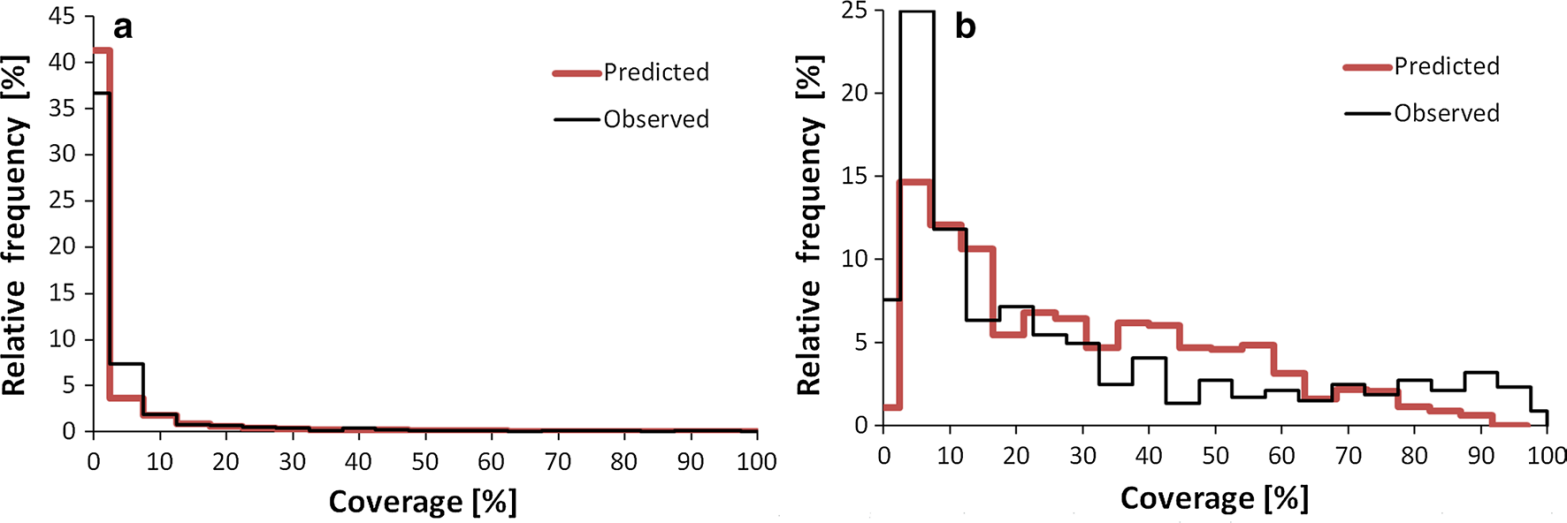
The frequency of *D. fruticosa* coverage values: **a** in the unclear presence/absence partition; **b** in the probable presence partition

The RMSE of coverage estimation for predicted presence sites in the unclear partition was 0.115 (with coverage as a continuous variable ranging from 0 to 1). Zero coverage dominated both the observed and predicted values (Fig. 10). In most cases, zero coverage was correctly predicted to be zero (specificity = 55.6%), which held the RMSE low.

**Fig. 11:**
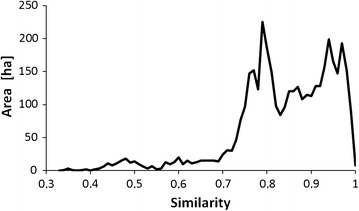
The frequency of similarity values when predicting *D. fruticosa* coverage

**Fig. 12:**
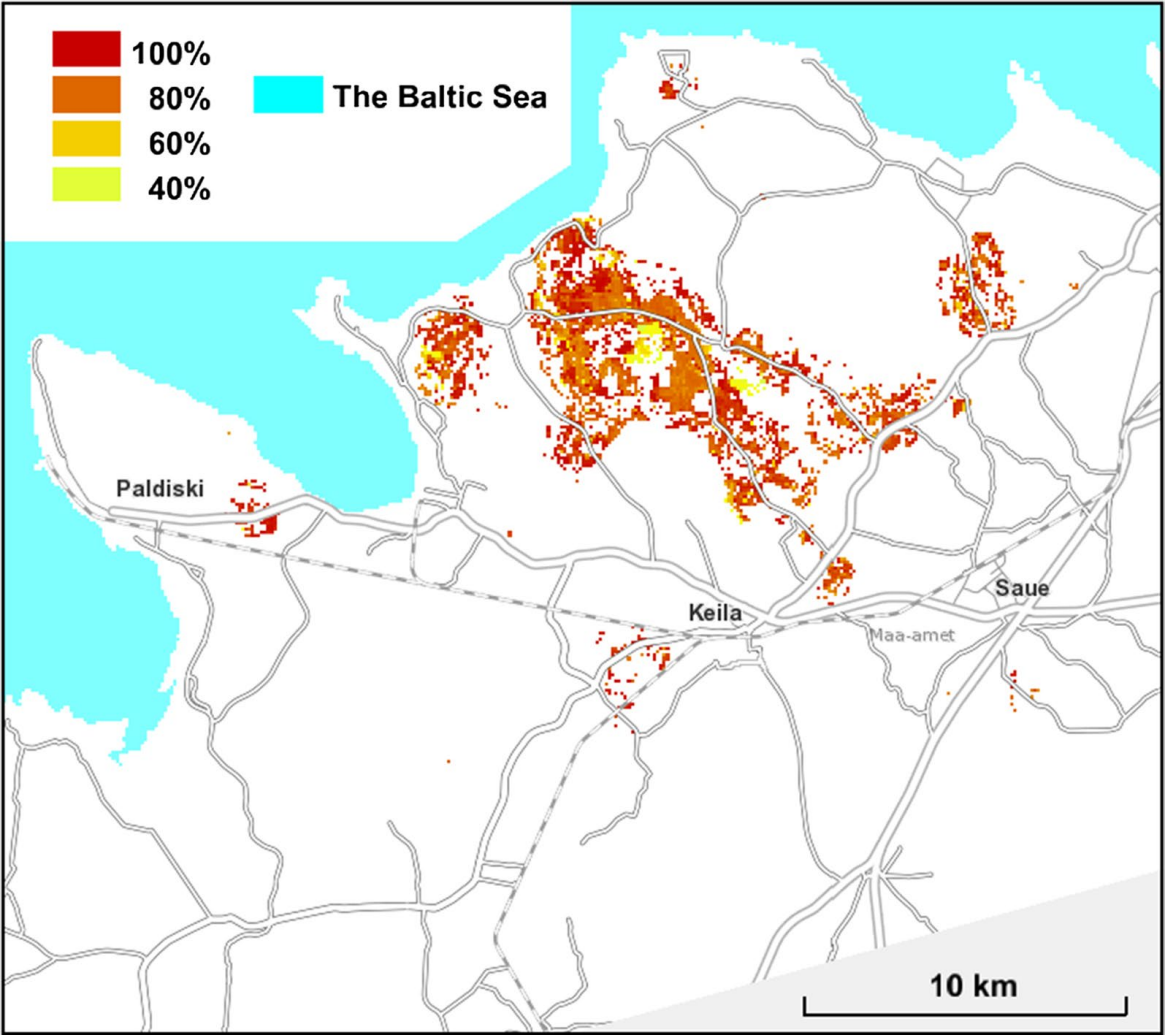
The similarity of locations to the exemplar locations used for predicting *D. fruticosa* coverage on a background map from the Estonian Land Board

The coverage estimation for the probable presence partition was much less reliable (RMSE = 0.244), despite all site features being included to the predictive set by machine learning in Constud. Species coverage in this partition was highly variable: there were 48 records among the thinned locations in the probable presence partition where *D. fruticosa* coverage reaches 0.9, together with 61 records of zero coverage. The mean similarity of predicted sites to exemplar sites used for *D. fruticosa* coverage mapping was mostly over 75% (Figs. 11, 12). Relatively low similarity values occurred on naturally rare or under-sampled land cover and soil types, e.g. on Floatic Histosol, which was represented by only two field records.

## Discussion

A novel software system, Constud 3, developed for similarity-based predictions, was used to map a fragmentally distributed clonal shrub around one of its core populations. A similarity-based approach to distribution mapping has rarely been used, and its implementation for abundance or plant coverage mapping is unknown to us. In their review, Elith and Leathwick [34], only denote similarity-based approaches in connection with species occurrence mapping, and not for numerical variables such as suitability or coverage. Predicting abundance is generally more complicated when compared to presence/absence, as a species’ abundance is unstable and depends more on population processes than habitat properties [38–40]. This study and some other publications [35–37] have however highlighted the ability of similarity-based methods to predict numerical variables.

The predictive map of *D. fruticosa* coverage (Fig. 8), demonstrates that the main protected area, created for the species, extends only over the main population core. However, most of the other populations and part of the core population are situated outside of protected areas. The distribution map could be applied for improving conservation planning and management. The coverage estimations could be used to find the most viable subpopulations, as well as to target management efforts, especially considering that dense *D. fruticosa* scrub shades out valuable herb layer of alvars, consequently resulting in a decrease in species richness [41].

In species distribution modelling approaches uncertainty generally increases near range edges, ecologically, due to increasing sensitivity to habitat conditions and, statistically, due to the procedure of converting probabilities from logistic regression into presence/absence [42]. In a more detailed scale of our study, the species actual presence or absence was remotely difficult to estimate (Fig. 9) in the population core area as the distribution is not continuous; the species absent locations are commonly near by the presence locations (Fig. 6).

In the case of coverage estimation, the RMSE was quite high—approximately equal to the mean), although the mean coverage of the species in the neighbourhood was included as predictive site feature. The high level of prediction error was not caused by excessive smoothing related to a high sum of similarity searched for decision, as the distribution of predicted values resembled the distribution of observed coverage values (Fig. 10b). It was more likely that the site features used did not adequately represent the spatial scale and real causes of variability in *D. fruticosa* coverage.

Spatial partitioning of a study area for separate models could be justified, if the area is diverse and each partition sufficiently covered by data. In most habitat suitability and species distribution studies, the study area is partitioned for stratified sampling [4]. Previously applied partitioning options for separate modelling have been based on site similarity [43], sampling effort (density of records) [44, 45], arbitrary geographical districts [46, 47], rings and quarters from the records distribution centroid [48], and random partitioning [49]. In the current study, as an innovation, separate predictive sets were used in spatial partitions formed according to the prior estimated expected presence/absence of the species. An additional option is to calibrate predictive sets separately for areas where one or another explanatory value is missing. For example, the soil data used in the current study contained void values, yet for similarity-based reasoning, data absence is not an obstacle, because the similarity level is calculated using existing site features. Splitting a study area complicates the predictive system but can produce more reliable results and is worth of comparative methodological studies.

The primary goal of modelling and similarity-based reasoning is to obtain predicted values for vague situations; predictions that confirm already well established knowledge are less useful. Therefore, data collection focused on possible, but unproven presence regions, and on less represented site types (rare land cover and soil categories in the current study), is less resource-consuming than performing spatially random or regular sampling. The latter would entail enormous time and resources spent in regions and habitat types unsuitable for the target species. Iterative adaptive observation effort is analogous to iterative boosting methods in machine learning [50], and to the construction of uncertainty or ignorance maps [51, 52]. This approach may reduce the formal goodness-of-fit of the predicted values in a learning sample, but is less inclined toward over fitting, i.e. it produces more reliable estimations outside of observed sites. However, a gradual redirection of observation effort to less represented site types, would lead to pseudoreplication, especially in the case of landscapes that are merely represented by single patches [53].

Equal and sufficient representation of all site types is difficult to achieve when using observations along transects or moving tracks. In the current study the number of thinned observation sites was as high as 9797, but some land cover and soil types were not represented by a sufficient number (1% of observations according to [26]) to prove the species absence. For example, although *D. fruticosa* was never observed growing in a bog, peat extraction site, or quagmire; these land cover types were not considered to indicate the species absence as containing less observations than necessary. Still, in case of a high location similarity to the exemplar presence locations regarding other features, some—likely false—presence predictions fell to those land cover types. E.g. *D. fruticosa* presence is predicted within the Tõlinõmme bog, which is situated in the middle of the species main population range, because of the ‘tussock area’ symbols on the historical map, which were also used to mark abundant *D. fruticosa* occurrence on alvars. In addition, frequent occurrence of the species around the bog supported the predicted occurrence of the species. If a detailed distribution mapping project is planned as a continuous process, the next field observations would primarily be directed to such places, followed by an update of the knowledge base.

The essence of similarity-based mapping, if compared to species niche modelling, is that the aim is not to find the environmental variables that result in the occurrence or absence of a species, but just to predict the distribution as accurately as possible. Short range spatial autocovariates can be effective predictors of dependent variables (especially for plants spreading by stems), but are applicable only near existing data. For example, proportion presences within 100 m was an effective predictor for a particular site, but for most of the study area, this site feature was useless, because it only covered 5.5% of the unclear presence/absence partition, despite the enormous amount of fieldwork undertaken (700 km on foot). Autocovariates calculated at a larger radius or as distance weighted, covered a larger proportion of the study area, but were less reliable predictors. Additional direct observations could add the close-neighbourhood area of the observed sites. Methodological alternatives for including neighbourhood effects, such as Markov’s chains, use the estimated and not the observed values of the target variable. The availability of estimated values is not limited by the extent of the neighbourhood, but these methods are computationally intensive and return unstable predictions [4].

## Conclusions

We encourage the use of similarity-based reasoning for habitat suitability and species distribution mapping, and recommend using the predictive map of *D. fruticosa* for conservation planning. We also suggest using separate models in different spatial partitions of any larger study area; and call others specialists to use the same data in connection with different methods in comparative methodological distribution mapping studies. The coordinates of observed sites and the recorded *D. fruticosa* coverages are freely available from doi:10.13140/RG.2.1.4987.6724. Contact the authors or apply a licence from the Estonian Land Board for using the topographical data layers.

## Abbreviations

Constud: a software system for similarity-based reasoning developed at the University of Tartu
GPS: Global Positioning System
ML: machine learning; in Constud software used in this project—iterative optimization of feature and case weights
RMSE: root mean squared error, the standard deviation of differences between observed and predicted values
SQL Server: a relational database management system developed by Microsoft

## References

[1] Guisan A, Zimmermann NE (2000). Predictive habitat distribution models in ecology. Ecol Model.

[2] Elith J, Graham CH, Anderson RP, Dudík M, Ferrier S, Guisan A (2006). Novel methods improve prediction of species’ distributions from occurrence data. Ecography.

[3] Austin MP (2007). Species distribution models and ecological theory: a critical assessment and some possible new approaches. Ecol Model.

[4] Franklin J (2009). Mapping species distributions. Spatial inference and prediction.

[5] Remm K (2004). Case-based predictions for species and habitat mapping. Ecol Model.

[6] Guisan A, Thuiller W (2005). Predicting species distributions: offering more than simple habitat models. Ecol Lett.

[7] Rodríguez JP, Brotons L, Bustamante J, Seoane J (2007). The application of predictive modelling of species distribution to biodiversity conservation. Divers Distrib.

[8] Aha DW (1998). The omnipresence of case-based reasoning in science and application. Knowl Based Syst.

[9] Chalom E, Asa E, Biton E (2013). Measuring image similarity: an overview of some useful applications. IEEE Instrum Meas Mag.

[10] Marling C, Montani S, Bichindaritz I, Funk P (2014). Synergistic case-based reasoning in medical domains. Expert Syst Appl.

[11] Choi D, Ko B, Kim H, Kim P (2014). Text analysis for detecting terrorism-related articles on the web. J Netw Comput Appl.

[12] Shokouhi SV, Skalle P, Aamodt A (2014). An overview of case-based reasoning applications in drilling engineering. Artif Intell Rev.

[13] Singh D, Ganju A, Singh A (2005). Weather prediction using nearest-neighbour model. Curr Sci India.

[14] Jasiewicz J, Netzel P, Stepinski TF (2014). Landscape similarity, retrieval, and machine mapping of physiographic units. Geomorphology.

[15] Booth TH (1990). A climatic analysis method for expert systems assisting tree species introductions. Agrofor Syst.

[16] Carpenter G, Gillison AN, Winter J (1993). DOMAIN: a flexible modelling procedure for mapping potential distributions of plants and animals. Biodivers Conserv.

[17] Clark JD, Dunn JE, Smith KG (1993). A multivariate model of female black bear habitat use for a geographic information system. J Wildlife Manag.

[18] Skov F (2000). Potential plant distribution mapping based on climatic similarity. Taxon.

[19] de Siqueira MF, Durigan G, de Júnior MP, Peterson AT (2009). Something from nothing: using landscape similarity and ecological niche modeling to find rare plant species. J Nat Conserv.

[20] Remm K, Remm L (2009). Similarity-based large-scale distribution mapping of orchids. Biodivers Conserv.

[21] Leht M, Reier Ü (1999). Origin, chromosome number and reproduction biology of *Potentilla fruticosa* (Rosaceae) in Estonia and Latvia. Acta Bot Fenn.

[22] Lonati M, Pascale M, Operti B, Lombardi G (2014). Synecology, conservation status and IUCN assessment of *Potentilla fr*uticosa L. in the Italian Alps. Acta Bot Gallica.

[23] Elkington TT (1969). Cytotaxonomic variation in *Potentilla fruticosa* L.. New Phytol.

[24] Pigott CD, Walters SM (1954). On the interpretation of the discontinuous distributions shown by certain British species of open habitats. J Ecol.

[25] Elkington TT (1963). Woodell SRJ. *Potentilla fruticosa* L. (*Dasiphora fruticosa* (L.) Rydb.). J Ecol.

[26] Remm K (2016). Selecting site characteristics at different spatial and thematic scales for shrubby cinquefoil (*Potentilla fruticosa* L.) distribution mapping. For Stud.

[27] Arold I. *Eesti Maastikud* (Estonian Landscapes). Tartu Ülikooli Kirjastus; 2005.

[28] Remm K. Shrubby cinquefoil (*Dasiphora fruticosa*) cover records from a study site in the NW Estonia. 2016. doi:10.13140/RG.2.1.4987.6724.

[29] Remm K, Remm M (2008). Case-based estimation of the risk of enterobiasis. Artif Intell Med.

[30] Remm K, Kelviste T. Constud Tutorial. University of Tartu; 2011.

[31] http://digiarhiiv.ut.ee/Constud3/. Accessed 23 July 2016.

[32] Gower JC (1971). A general coefficient of similarity and some of its properties. Biometrics.

[33] Allouche O, Tsoar A, Kadmon R (2006). Assessing the accuracy of species distribution models: prevalence, kappa and the true skill statistic (TSS). J Appl Ecol.

[34] Elith J, Leathwick JR (2009). Species distribution models: ecological explanation and prediction across space and time. Annu Rev Ecol Evol Syst.

[35] Gayer G, Gilboa I, Lieberman O. Rule-based and case-based reasoning in housing prices. BE J Theor Econ 2007;7.

[36] Liu F, Rossiter DG, Song X-D, Zhang G-L, Yang R-M, Zhao Y-G, Li D-C, Ju B (2014). A similarity-based method for three-dimensional prediction of soil organic matter concentration. Geoderma.

[37] Yang L, Huang C, Liu G, Liu J, Zhu A-X (2015). Mapping soil salinity using a similarity-based prediction approach: a case study in Huanghe River Delta, China. Chin Geogr Sci.

[38] van Horne B (1983). Density as misleading indicator of habitat quality. ‎J Wildl Manag.

[39] Frescino TS, Edwards TC, Moisen GG (2001). Modelling spatially explicit forest structural attributes using generalized additive models. J Veg Sci.

[40] Pearce J, Ferrier S (2001). The practical value of modelling relative abundance of species for regional conservation planning: a case study. Biol Conserv.

[41] Rejmánek M, Rosén E (1988). The effects of colonizing shrubs (*Juniperus communis* and *Potentilla fruticosa*) on species richness in the grasslands of Stora Alvaret, Öland (Sweden). Acta Phytogeographica Suecica.

[42] Hanspach J, Kühn I, Schweiger O, Pompe S, Klotz S (2011). Geographical patterns in prediction errors of species distribution models. Global Ecol Biogeogr.

[43] Estrada-Peña A, Thuiller W (2008). An assessment of the effect of data partitioning on the performance of modelling algorithms for habitat suitability for ticks. Med Vet Entomol.

[44] Fourcade Y, Engler JO, Besnard AG, Rödder D, Secondi J (2013). Confronting expert-based and modelled distributions for species with uncertain conservation status: a case study from the Corncrake (*Crex crex*). Biol Conserv.

[45] Fourcade Y, Engler JO, Rödder D, Secondi J (2014). Mapping species distributions with MAXENT using a geographically biased sample of presence data: a performance assessment of methods for correcting sampling bias. PLoS ONE.

[46] Murphy HT, Lovett-Doust J (2007). Accounting for regional niche variation in habitat suitability models. Oikos.

[47] Gonzalez SC, Soto-Centeno JA, Reed DL (2011). Population distribution models: species distributions are better modeled using biologically relevant data partitions. BMC Ecol.

[48] Osborne PE, Suárez-Seoane S (2002). Should data be partitioned spatially before building large-scale distribution models?. Ecol Model.

[49] Phillips SJ, Anderson RP, Schapire RE (2006). Maximum entropy modeling of species geographic distributions. Ecol Model.

[50] Freund Y, Schapire RE (1997). A decision-theoretic generalization of on-line learning and an application to boosting. J Comput Syst Sci.

[51] Rocchini D, Hortal J, Lengyel S, Lobo JM, Jiménez-Valverde A, Ricotta C (2011). Accounting for uncertainty when mapping species distributions: the need for maps of ignorance. Progr Phys Geogr.

[52] Beale CM, Lennon JJ (2012). Incorporating uncertainty in predictive species distribution modelling. Phil Trans R Soc B.

[53] Hurlbert SH (1984). Pseudoreplication and the design of ecological field experiments. Ecol Monogr.

